# Aggregation Controlled Charge Generation in Fullerene Based Bulk Heterojunction Polymer Solar Cells: Effect of Additive

**DOI:** 10.3390/polym13010115

**Published:** 2020-12-30

**Authors:** Washat Ware, Tia Wright, Yimin Mao, Shubo Han, Jessa Guffie, Evgeny O. Danilov, Jeromy Rech, Wei You, Zhiping Luo, Bhoj Gautam

**Affiliations:** 1 Department of Chemistry, Physics and Materials Science, Fayetteville State University, Fayetteville, NC 28301, USA; wware2@broncos.uncfsu.edu (W.W.); twrigh38@broncos.uncfsu.edu (T.W.); shan@uncfsu.edu (S.H.); jguffie@broncos.uncfsu.edu (J.G.); zluo@uncfsu.edu (Z.L.); 2 NIST Center for Neutron Research, National Institute of Standards and Technology, 100 Bureau Drive, Gaithersburg, MD 20899, USA; yimin.mao@nist.gov; 3 Department of Materials Science and Engineering, University of Maryland, College Park, MD 20742, USA; 4 Department of Chemistry, North Carolina State University, Raleigh, NC 27695, USA; danilov@ncsu.edu; 5 Department of Chemistry, University of North Carolina at Chapel Hill, Chapel Hill, NC 27599, USA; jeromy@live.unc.edu (J.R.); wyou@unc.edu (W.Y.)

**Keywords:** charge, exciton, aggregation, polymer solar cells, morphology

## Abstract

Optimization of charge generation in polymer blends is crucial for the fabrication of highly efficient polymer solar cells. While the impacts of the polymer chemical structure, energy alignment, and interface on charge generation have been well studied, not much is known about the impact of polymer aggregation on charge generation. Here, we studied the impact of aggregation on charge generation using transient absorption spectroscopy, neutron scattering, and atomic force microscopy. Our measurements indicate that the 1,8-diiodooctane additive can change the aggregation behavior of poly(benzodithiophene-alt-dithienyl difluorobenzotriazole (PBnDT-FTAZ) and phenyl-C61-butyric acid methyl ester (PCBM)polymer blends and impact the charge generation process. Our observations show that the charge generation can be optimized by tuning the aggregation in polymer blends, which can be beneficial for the design of highly efficient fullerene-based organic photovoltaic devices.

## 1. Introduction

Organic semiconductors (OSCs) have been intensively studied due to their unique electronic and optical properties. Their properties—including relatively easy and inexpensive fabrication, light weight, mechanical flexibility and compatibility with stretchability, and potential for non-toxic processing methods—open broad prospects for their applications in a variety of industrial and technological areas, including solar cells [1,2]. Considerable efforts have been dedicated to the development of polymer solar cells (PSCs) due to several advantages, such as high absorption coefficients [3], highly tunable molecular energy levels [4], and low reorganization energy associated with low voltage loss [5,6]. To date, power conversion efficiency of over 17% [7] has been achieved in PSCs. There are several factors that influence charge generation and transport in bulk heterojunction (BHJ) polymer solar cells. These include the miscibility of donor and acceptors [8], molecular orientation of donor and acceptors at the interface [9], energy difference between the bulk excitonic states and interfacial charge transfer (CT) states [10,11], domain size [12], and the interaction between donor and acceptors [13,14]. In addition, the molecular order [15] and packing [16,17] determine the electronic interactions [18], which influence, for instance, exciton delocalization [19] and charge generation [20]. Furthermore, change in morphology can impact processes such as charge generation [21], charge transport [22], and optical absorption and emission [23,24]. It has been reported that the addition of solvent additives such as 1,8-diiodoctane (DIO) in polymer blends results in the change in nanomorphology of the BHJ active layer [25,26]. The improved morphology by DIO resulted in the high charge transfer and charge transport efficiency that are needed for high-efficiency PSCs. Therefore, it is very important to understand how the molecules assemble in thin BHJ films and how the species of the assemblies affect the solar cell efficiency.

As the charge carrier motion in polymer films depends on the coupling of the electronic states, the electronic coupling in these systems has direct implications for the charge generation and transport; hence, it impacts the device performance [23]. When the molecular arrangement leads to the transition dipole moments interacting along the polymer backbone, strong intrachain electronic coupling is obtained, which is referred to as J-like, in analogy to J-aggregates in molecular systems [23,27]. In contrast, parallel π-π packing of multiple chains favors strong interchain electronic coupling, referred to as H-like [23,24,28]. Therefore, it is crucial to understand the impact of electronic coupling on the optoelectronic properties of these materials for their applications in photovoltaic devices.

In this work, we prepared thin blended films from medium-band gap copolymer poly(benzodithiophene-alt-dithienyl difluorobenzotriazole (PBnDT-FTAZ) [29] and phenyl-C61-butyric acid methyl ester (PCBM). The PBnDT-FTAZ polymer consists of a benzodithiophene (BnDT) donor moiety and fluorinated benzotriazole (FTAZ) acceptor moiety. This donor polymer has shown planar conformation, molecular face-on orientation, and high hole mobility [9,29]. The acceptor PCBM has (i) high electron mobility, (ii) ability to aggregate in BHJ, and (iii) good charge transport due to a delocalized lowest unoccupied molecular orbital over the entire surface of the molecule [30]. These properties of donor and acceptor molecules are desired for the fabrication of highly efficient PSCs. In this contribution, we used a PBnDT-FTAZ:PCBM blend with and without the solvent additive DIO to investigate the impact of DIO on aggregation, which can modify the optical absorption and emission spectra in polymer blends. Using transient absorption spectroscopy (TAS), small-angle neutron scattering (SANS), and atomic force microscopy (AFM), we probed the changes in aggregation, charge dynamics, exciton delocalization, and the morphology. Our observations indicate that the electronic coupling in conjugated polymer blends can be tuned by processing methods and can be probed using optical absorption and emission measurements.

## 2. Materials and Methods

### 2.1. Materials

PBnDT-FTAZ polymer was synthesized by Prof. Wei You’s lab at the University of North Carolina, Chapel Hill in the same way as previously reported [29]. PCBM and DIO additive were purchased from Sigma Aldrich and were used as received.

### 2.2. Thin Film Preparation

PBnDT-FTAZ:PCBM blend solution (20 mg/mL) was prepared in chlorobenzene with a donor/acceptor weight ratio of 1:2. The blend solution was heated at 80 °C and stirred overnight before mixing with DIO (3 wt.%, defined as percentage of total PBnDT-FTAZ:PCBM weight) and stirred for one additional hour. Glass substrates were cleaned ultrasonically using DI water, acetone, and isopropanol for 15 min per cleaning solvent before spin casting. Blend films with and without DIO were prepared by spin casting the hot solution onto the precleaned glass substrates at 500 rpm for 60 s. The thin film samples were encapsulated using UV curable glue before optical measurements [31]. Thin films for SANS were prepared by casting the solution in one-inch diameter silicon wafer. All the thin films were prepared inside a nitrogen filled glove box.

### 2.3. Absorption and Photoluminescence Measurements

A Cary 100 UV-spectrophotometer from Varian was used for the ground state absorption measurement in the spectral range 350–900 nm, which was carried out at ambient conditions. The room temperature, steady state photoluminescence (PL) spectra in the UV-NIR spectral range were recorded using an Edinburgh Instruments Fluorescence Spectrometer (Model: FLS900) equipped with a xenon lamp (Xe 900, xenon arc lamp). The samples were excited with 2.48 eV (500 nm) excitation energy and the emitted PL was detected using the red sensitive PMT [31].

### 2.4. Transient Absorption Spectroscopy

Transient absorption spectroscopy (TAS) was measured using an Ultrafast Systems Helios pump-probe transient absorption spectrometer. Coherent Libra Ti:Sapphire femtosecond regenerative amplifier (4 W, 1 kHz, 800 nm, 100 fs) was used as a source for both pump and probe pulses. The output of the amplifier was split into two beams. The first beam pumped a Coherent OperA Solo optical parameter amplifier which converted the 800 nm input to a 2.48 eV (500 nm) output. We kept the pump excitation intensity low (~2 μJ/cm^2^) to avoid possible exciton-exciton annihilation and non- linear effects. The second beam generated a broadband white light continuum (WLC) from 0.8 eV to 1.55 eV by focusing 800 nm light into a sapphire plate. The pump and probe beams were then overlapped spatially on the thin film sample. The WLC transmitted through the sample was sent to a fiber optics coupled linear array spectrometer. The pump-probe delay was controlled by an optical line with a range of approximately 5 ns [31].

### 2.5. AFM Measurements

AFM topographic images and phase images were taken using the AAC mode with a Keysight 5500 AFM/SPM system (Keysight Technologies, Inc., CO, USA). A Bruker’s Sharp Nitride Lever probe, SNL-10, with a normal frequency of 65 kHz and a normal spring constant of 0.35 N/m was used in the AFM scanning (Bruker AFM Probes, Camarillo, CA 93012, USA).

### 2.6. SANS Measurements

Small-angle neutron scattering experiments were carried out at the NGB 30 m SANS beamline at the NIST Center for Neutron Research (NCNR), National Institute of Standards and Technology (NIST) [32]. Five instrumental configurations were used to collect SANS data from q≈0.001 to $q\approx 1.0\;{Å}^{-1}$ (q was the momentum transfer defined as q=4πsinθ/λ, with θ and λ being half of the scattering angle and neutron wavelength, respectively). Neutron wavelength was 6 Å (wavelength spread $\frac{\Delta \lambda }{\lambda }\approx 14\%$) at the sample-to-detector-distances (SDDs) of 1 m, 4 m, and 13 m, which covers a *q*-range between q≈0.003 and $q\approx 0.5\;{Å}^{-1}$. Low- q scattering data extended to $q\approx 0.001\;{Å}^{-1}$ (was collected using a focused neutron beam at λ≈8.4 Å. High- q (between q≈0.5 and $q\approx 1.0\;{Å}^{-1}$.) scattering data were collected using 3 wavelength at a broader wavelength spread of ≈22%).

To obtain scattering from polymer films, scattering data from polymer films deposited on silicon wafers and that from a blank wafer were measured separately, and then the scattering from the blank wafer were subtracted with transmission coefficients of the samples being handled properly. All 1D scattering profiles have been normalized to absolute scale. Details of data reduction protocol can be found in Ref. [33].

## 3. Results and Discussion

**Figure 1:**
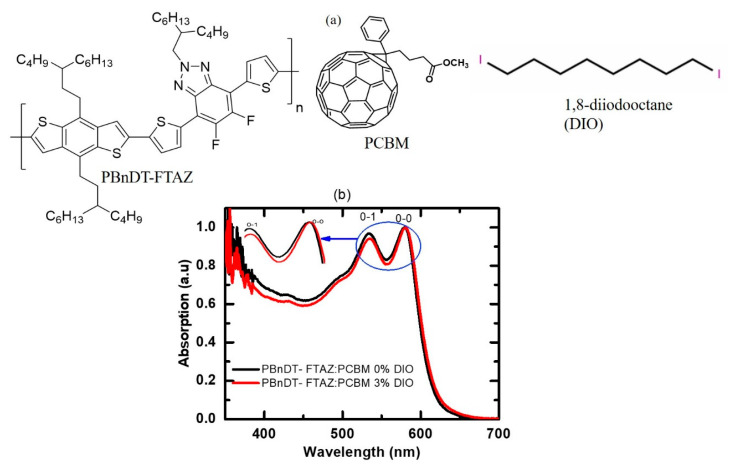
(**a**) Chemical structures of PBnDT-FTAZ, PCBM and DIO, and (**b**) absorption spectra of PBnDT-FTAZ:PCBM with and without DIO.

Figure 1a shows the chemical structure of the donor PBnDT-FTAZ polymer, electron acceptor PCBM molecule, and DIO additive, whereas Figure 1b shows the absorption spectra of PBnDT-FTAZ:PCBM with and without DIO. Vibronic features below 650 nm are reflected in the absorption spectra of both films. The intensities of the first two vibronic peaks attributed as 0–0 and 0–1 transitions in the absorption spectra identify the different types of aggregation [26]. When the 0–0 absorption is stronger than that of the 0–1 transition, the polymer is J-like, and when the reverse is the case, the material is H-like aggregated. The DIO additive leads to slight differences in aggregation of PBnDT-FTAZ:PCBM, which is reflected in the relative intensities of the 0–0 and 0–1 vibronic transitions in the absorption spectra. The ratio of intensity of the 0–0 peak to 0–1 is 1.03 for the pristine PBnDT-FTAZ:PCBM blend, whereas it increases to 1.06 for the DIO-added blend peak, suggesting J-like aggregation in both blends [28,34]. However, as the ratio of 0–0 to 0–1 vibronic peaks increases with the addition of DIO, this suggests that the additive can induce more J-aggregated behavior in the PBnDT-FTAZ:PCBM films. The slightly red-shifted absorption in DIO-added blend further supports this assignment [24]. It has been reported that PBnDT-FTAZ also shows more J-like behavior when it is blended with non-fullerene acceptor and DIO is added [35]. The observed strong J-aggregation leads to a stronger intrachain exciton coupling, planarization of the polymer backbone, enhanced crystallinity, and a higher hole mobility [24,36]. The more J-like absorption characteristics in the DIO-added blend are attributed to: (i) conformational changes, which may increase the planarity of the polymer backbone, and (ii) a higher delocalization of the π -electron density along the more planarized conjugation system, which may result in enhanced intrachain exciton interactions [24]. These subtle conformational differences have direct consequences on the head-to-tail interactions of the transition dipole moments of the chromophores, which ultimately influence the spectral line shapes [37].

The ratio of 0–0 and 0–1 peak absorbance is related to the free exciton bandwidth (*W*) [38] and the energy of the intermolecular vibration *E_p_*. We calculated *W* of these blended films using weakly interchain-coupled modified Frank-Condon model [28,39], $\frac{A_{0-0}}{A_{0-1}}\approx {\left(\frac{1-\frac{0.24W}{E_{p}}}{1+\frac{0.73W}{E_{p}}}\right)}^{2}$, where $A_{0-0}$ and $A_{0-1}$ are peak absorbances from Figure 1b, and *E_p_* was obtained from the difference in energy of 0–0 and 0–1 absorbance peaks. We obtained the exciton bandwidth (*W*) of −8.60 meV and −16.9 meV for the blend films with and without DIO, respectively. The negative *W* further indicates the J-aggregate molecular packing in these blends [28]. The different exciton bandwidths of these two blends indicate that DIO addition does, in fact, alter the conjugation length of the polymer chain. In reality, both coupling mechanisms are present in conjugated polymers, as described by the generalized HJ aggregation model [18,40]. It is noted that the H- and J-aggregate analysis of steady-state polymer UV-Vis spectra has provided significant insights into the interplay between structural and optoelectronic properties of polymers and unraveled some of the physics behind the differences in measured spectral line shapes in solution, as well as the amorphous and aggregated fractions of the material in the solid state [28,34,41].

**Figure 2:**
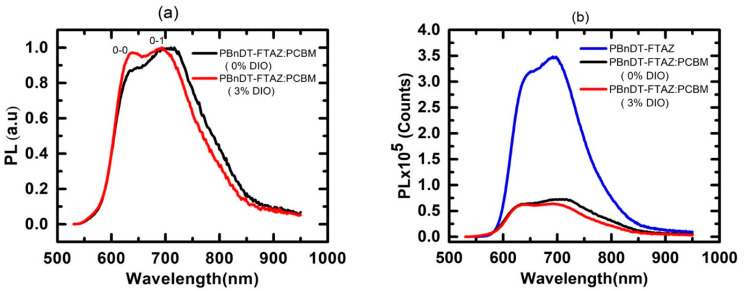
(**a**) Normalized Photoluminescence spectra of PBnDT-FTAZ:PCBM with and without DIO (**b**) photoluminescence spectra of PBnDT-FTAZ neat film, and blended films with and without DIO.

In contrast to the absorption spectra, the steady state PL spectrum of the pristine blend and DIO added blend films do not exhibit J-like character. The 0–1 emission is stronger compared to the 0-0 transition (Figure 2a). The ratio of intensity of 0–0 peak to 0–1 is 0.88 and 0.97 for pristine PBnDT-FTAZ:PCBM and DIO added blend films respectively. This difference, thus, suggests that the photo-excited species created in the absorption process and those that recombine during the emission exhibit very different intra- and interchain electronic interactions because there is a change in electronic coupling during the relaxation process [42]. Spano et al. established the connection between the vibronic progression and exciton delocalization through the ratio 0–0 to 0–1 photoluminescence intensity in molecular aggregates [43]. This ratio is proportional to the exciton coherence length [44], which is related to exciton delocalization [43]. Therefore, there can be slight differences in the 0–0/0–1 ratios in the absorption and emission lines, because the PL is more sensitive to the exciton coherence length, while the absorption is more sensitive to the free exciton bandwidth. As a result, it is common to observe stronger characteristics of one type over the other when comparing absorption and emission profiles. In general, though, within the Spano model, the emission is not completely symmetrical with the absorption [23].

The spectroscopic measurements, such as ground-state absorption and PL, provided the information about the effect of DIO in polymer aggregation and exciton delocalization in PBnDT-FTAZ:PCBM. To understand the interrelation between the exciton delocalization and the charge transfer, we measured the PL of the neat PBnDT-FTAZ and blended films. Comparison of the PL between the blended and the neat films indicates quenching of polymer photoluminescence. We observed the 73% PL-quenching efficiency in the pristine blend, whereas it is 78% in the DIO-added blend (Figure 2b). The increased PL-quenching efficiency in the DIO-added blend suggests the increase in charge separation in this blend [45,46]. The PL quenching is the indicator of the exciton splitting at the donor–acceptor interface and provides the indication of an upper limit to the yield of dissociated charges [46]. As the PL is insensitive to the non-radiative species, such as polaron pairs, charges, etc., we utilized TAS—a widely used technique that is crucial for detecting non-luminescent species, such as polarons/charges or polaron pairs, and their time evolution.

**Figure 3:**
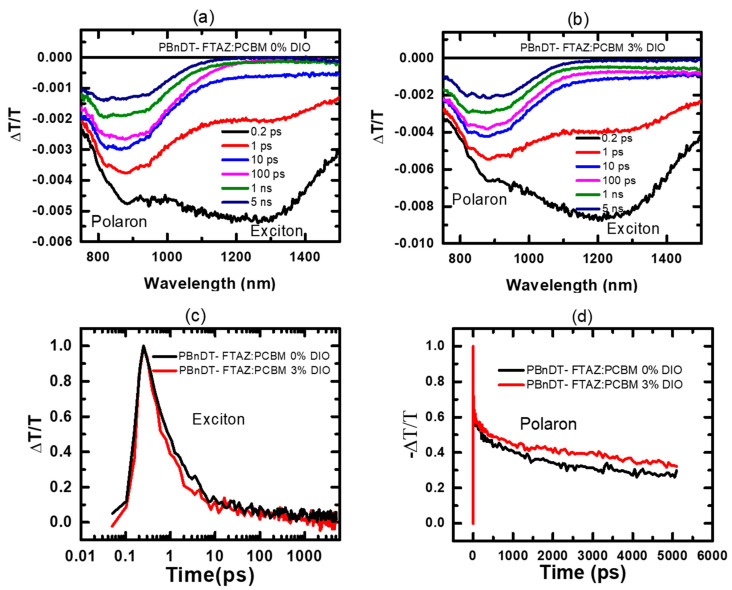
Transient absorption spectra of PBnDT-FTAZ:PCBM blend films with (**a**) 0% DIO and (**b**) 3% DIO, and comparation of (**c**) exciton and (**d**) charge separation dynamics.

Figure 3 shows the TAS spectra, the exciton, and charge (polaron) separation dynamics of pristine and DIO added PBnDT-FTAZ:PCBM blends. Figure 3a,b show the transient absorption spectra at different time delays after the samples are excited using pump pulses tuned to 2.48 eV (500 nm). The transient absorption spectrum of both blends exhibits two features at ~1250 nm and ~880 nm. Based on the previously published results on conjugated polymers, we assign these two peaks to the excited-state absorption of the polymer singlet exciton and polaron, respectively [10,47,48,49]. Exciton and polaron dynamics were monitored by plotting the time evolution of the features at ~1250 nm and ~880 nm. The averaged exciton lifetimes, obtained from double exponential function, are 4.1 ps and 2.6 ps for the pristine and the DIO added blend, respectively, indicating that the electron transfer from PBnDT-FTAZ to PCBM is more favored in the DIO added blend (Figure 3c). The polaron lifetimes for pristine and the DIO added blend are 365 ps and 420 ps with 30% and 35% respective residual charges at 5 ns (Figure 3d). These results indicate the efficient electron transfer and long-lived charge generation in the DIO added blend. These observations are consistent with the improved fill factor and higher short circuit current density observed in the 3% DIO added PBnDT-FTAZ:PCBM solar cell device compared to that of 0% DIO PBnDT-FTAZ:PCBM device [45].

**Figure 4:**
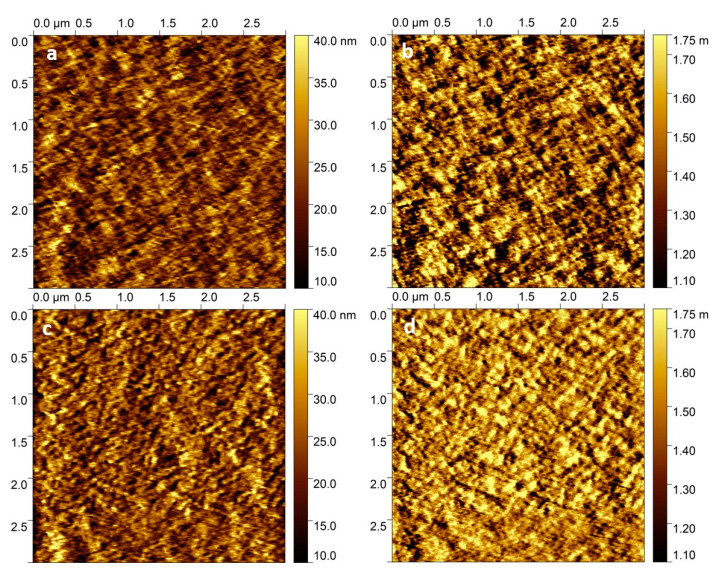
AFM topography (**a**,**c**) and phase (**b**,**d**) images of PBnDT-FTAZ:PCBM blend films with 0% (**a**,**b**) and 3% DIO (**c**,**d**).

To verify that the changes in optical properties are microstructural in origin, we studied the surface morphology of these thin films using AFM. Figure 4 shows the AFM topographic and phase images of PBnDT-FTAZ:PCBM blend films with 0% and 3% DIO. We observed morphological changes in the PBnDT-FTAZ:PCBM blend upon the addition of DIO. Specifically, under the same AFM scanning conditions, the 3% DIO-added blend formed a firmer surface with less drifting (Figure 4c,d) than that of the PBnDT-FTAZ:PCBM blend without DIO (Figure 4a,b) in the AFM images. Statistical analysis showed a 0.84 µm^2^ surface area increment for the 3% DIO-added blend compared to the only 0.61 µm^2^ increment for the 0% DIO-added blend at a 9 µm^2^ range, with a significant 37.7% increase. The phase image median root mean square (RMS) was measured to be 0.14 deg for the 3% DIO-added and 0.024 deg for the 0% DIO-added (Table 1). These facts suggest an increased phase separation between the PBnDT-FTAZ polymer and PCBM and more rigid aggregates formed after adding 3% DIO—a desired effect for efficient charge generation and transport. These observations are consistent with optical spectroscopy data, which show more exciton delocalization, efficient electron transfer, and long-lived charges in the DIO-added PBnDT-FTAZ:PCBM blend.

**Figure 5:**
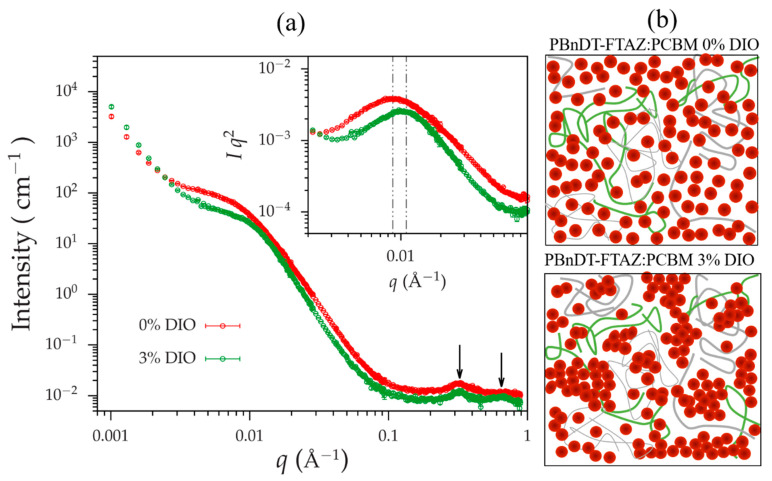
(**a**) 1D SANS profiles of films of PBnDT-FTAZ: PCBM with (3%) and without DIO and (**b**) Schematic of hierarchical structures in those blends.

We extended our morphological study by performing SANS measurements. Figure 5a shows SANS profiles of PBnDT-FTAZ: PCBM films with and without DIO. The SANS profiles are distinctly different in three aspects. First, in the high- *q* region between $q\approx 0.2\;{Å}^{-1}$ and $q\approx 1.0\;{Å}^{-1}$, two scattering maxima can be clearly observed for the film with 3% of DIO. Those are Bragg peaks originated from the close packing of the PCBM particles. The peaks intrinsically are broad due to the order of packing and to the instrumental broadening (particularly associated with the neutron wavelength spread). Nevertheless, the positions of the first and the second peaks can be approximately identified at q≈0.33 and $q\approx 0.65\;{Å}^{-1}$ which is consistent with reported body-centered cubic (BCC) packing [50], allowing uncertainties associated with the broad peaks.

The second peak was absent in the scattering profile from the sample without DIO, which might be due to the more severe disorder of packing, given that the two samples were measured using the same instrumental configurations. Second, a broad ‘shoulder’ shows in the intermediate- *q* region between q≈0.004 and $q\approx 0.02\;{Å}^{-1}$ for both the profiles. The ‘shoulder’ is a manifestation of interference of waves scattered from nanoscale domains. Note that for the polymer film with 3 % of DIO, the ‘shoulder’ shifts toward higher- *q*, suggesting that the domain is smaller as compared with that of the film without DIO. The slight change of the position of the ‘shoulder’ can be clearly seen in the $Iq^{2}$ versus q plot (in-set of Figure 5a), where the maxima are at ≈0.0087 and $q\approx 0.011\;{Å}^{-1}$, respectively, for the samples without and with 3 % DIO. This is corresponding to a shrinkage of domain size from ≈7.2 nm to ≈5.7 nm (estimated using, $2\pi /q_{m}$ with $q_{m}$ being the position of the maximum in the $Iq^{2}$ versus q plot), owing to the addition of DIO. Third, the two profiles show scattering upturns in the region of $q<\approx 0.02\;{Å}^{-1}$ for both profiles. The upturns phenomenologically follow a power-law decay, and the sample with 3 % DIO shows a larger asymptote, which suggests the existence of larger macroscopic aggregates with the sizes being out of the probe limit of SANS.

Combining all the observations, hierarchical structures in the PBnDT-FTAZ:PCBM films can be assessed, which is schematically shown in Figure 5b. PCBM particles are dispersed in polymer matrix, forming nanoscale domains. DIO can promote close packing of PCBM, which causes two consequences. On one hand, the PCBM domain size is smaller in the polymer film with DIO, as compared with that without DIO. On the other hand, the domains with more closely packed PCBM tend to form aggregates at an even larger length scale [51,52].

## 4. Conclusions

In this work, we prepared the pristine and DIO-added PBnDT-FTAZ:PCBM blend films to investigate the role of 1,8-diiodooctane additive on optical properties. We observed the changes in aggregation, exciton delocalization, electron transfer efficiency from donor to acceptor, and charge generation when 3% DIO was added to the pristine blend solution. Ground-state absorption and PL measurements indicate longer conjugation length and more exciton delocalization in DIO-added PBnDT-FTAZ:PCBM, whereas higher charge separation ability at the interface was observed in this blend in the TAS measurement. In addition, the AFM and SANS data indicate the greater phase separation and the aggregate formation in this blend. Our work indicates that the molecular conformation and aggregation changes caused by the DIO additive can result in the contrast in the device performance of fullerene-based PSCs. This suggests that understanding and controlling the microstructures of polymer-blend films using additives is important for optimizing the performance of PSCs.

## Figures and Tables

**Table 1:** Summary of Statistical Quantities of AFM Images.

Sample	Statistical Analysis
PBnDT-FTAZ: PCBM Blend Film with 0% DIO	Surface Area: 9.611 µm^2^; Projected Area: 9.000 µm^2^; RMS of Phase Image: 0.024 Deg
PBnDT-FTAZ: PCBM Blend Film with 3% DIO	Surface Area:9.844 µm^2^; Projected Area: 9.000 µm^2^; RMS of Phase Image: 0.14 Deg
